# The central role of mitochondrial pathology in sepsis-induced cardiomyopathy: from molecular mechanisms to clinical translation

**DOI:** 10.3389/fcvm.2026.1856434

**Published:** 2026-07-29

**Authors:** Linghong Xu, Jun Zhang, Huijing Tong, Xusheng Teng

**Affiliations:** Department of Emergency, Jinhua Municipal Central Hospital, Jinhua, China

**Keywords:** inflammation, mitochondrial dynamics, mitochondrial dysfunction, mitophagy, oxidative stress, sepsis-induced cardiomyopathy, therapeutic targets

## Abstract

Sepsis-induced cardiomyopathy (SIC) affects approximately 50% of severe sepsis patients, with mortality rates approaching 80%. This review examines mitochondrial pathology as the central orchestrator of SIC progression. Mitochondrial dysfunction encompasses impaired oxidative phosphorylation (OXPHOS), causing bioenergetic failure; mitochondrial DNA (mtDNA) release activating cyclic guanosine monophosphate-adenosine monophosphate synthase-stimulator of interferon genes, Toll-like receptor 9, and NOD-like receptor family pyrin domain containing 3 pathways; ETC dysfunction generating explosive reactive oxygen species (ROS); defective mitophagy leading to damaged mitochondria accumulation; and disturbed mitochondrial dynamics with excessive fission and suppressed fusion. Intercellular mitochondrial transfer through tunneling nanotubes (TNTs) exhibits paradoxical dual effects. Mitochondria-targeted antioxidants selectively accumulate within mitochondria to scavenge ROS and preserve membrane potential. Nrf2 activators enhance endogenous antioxidant defenses. Melatonin modulates mitochondrial function through Ripk3 inhibition. Clinical translation faces substantial obstacles due to sepsis heterogeneity, animal model limitations, and disease complexity. This review integrates mitochondrial biology, immunometabolism, and translational medicine to identify promising directions for improving patient outcomes.

## 1 Introduction

Sepsis is defined as life-threatening organ dysfunction resulting from a dysregulated host response to infection (1). Among the various organ dysfunctions that characterize this syndrome, cardiac injury represents the most fatal manifestation, significantly impacting patient prognosis. Sepsis-induced cardiomyopathy (SIC) constitutes a frequent complication of severe sepsis, developing in approximately 50% of patients, with associated mortality rates of approximately 80% (2). The management of SIC imposes considerable economic burdens and extensive healthcare resource utilization, markedly compromising patients’ quality of life. Despite advances in critical care, effective therapeutic strategies for SIC remain limited, necessitating a deeper understanding of its underlying pathophysiological mechanisms.

Recent research has yielded significant insights into the complex pathogenesis of SIC. The condition is characterized by both systolic and diastolic dysfunction, with myocardial injury resulting from hemodynamic alterations, cardiomyocyte death through apoptosis and autophagy, calcium dyshomeostasis, and excessive inflammatory responses (3–5). Notably, emerging evidence highlights the central role of mitochondrial dysfunction in SIC pathophysiology. Mitochondria, as the principal energy generators of eukaryotic cells, regulate not only ATP synthesis but also calcium homeostasis, ROS generation, and programmed cell death pathways (6, 7). In sepsis, mitochondrial damage leads to bioenergetic failure, oxidative stress bursts, and the release of mitochondrial damage-associated molecular patterns (DAMPs), which perpetuate inflammatory responses and exacerbate cardiac dysfunction (8, 9).

Given the pivotal involvement of mitochondria in SIC, targeted interventions aimed at preserving mitochondrial function have attracted considerable attention as potential therapeutic strategies. These include mitochondria-targeted antioxidants, modulators of mitochondrial dynamics, and agents that enhance mitophagy (10, 11). However, the translation of these promising preclinical findings into clinical practice faces substantial challenges due to the heterogeneity and complexity of human sepsis (12).

This review provides a comprehensive overview of the pathogenesis of SIC, with particular emphasis on mitochondrial dysfunction as the central pathogenic driver. We systematically examine the mechanisms underlying mitochondrial dysfunction in SIC, including impaired OXPHOS, aberrant mitochondrial dynamics, defective mitophagy, and mitochondrial-mediated inflammatory responses. Furthermore, we discuss current therapeutic strategies targeting mitochondria and address the challenges in clinical translation, aiming to identify promising directions for future research and treatment development.

## 2 Pathogenesis of SIC

Cardiac injury represents the most fatal manifestation among these organ dysfunctions. SIC constitutes a frequent complication of severe sepsis, developing in approximately 50% of patients, with associated mortality rates of approximately 80% (2), thereby constituting a grave threat to patient prognosis. The management of SIC imposes considerable economic burdens and extensive healthcare resource utilization, markedly compromising patients’ quality of life. Recent advances in SIC research have yielded significant insights into its underlying pathophysiological mechanisms and emerging therapeutic approaches.

### 2.1 Hemodynamic and myocardial alterations

Myocardial injury in septic patients is frequently accompanied by both systolic and diastolic dysfunction. Beesley et al. (3) observed in a study of 67 patients with septic shock that approximately 20% developed SIC within 6 h of onset, with the incidence increasing to over 60% by 1–3 days, a progression potentially attributable to early peripheral vasodilation, left ventricular compensation, and disease advancement in sepsis. Another study demonstrated that septic patients exhibited impaired cardiac contractility, manifesting as reduced cardiac index and ejection fraction; although overall hemodynamics remained stable, regional alterations in myocardial blood flow could lead to local hypoperfusion, ischemia, and necrosis. Concurrently, sepsis-induced decreased coronary artery compliance and ventricular elastance ultimately resulted in diminished contractility (4). Lu et al. (5) further reported that in a lipopolysaccharide (LPS)-induced sepsis mouse model, myocardial fibers appeared disorganized, with focal myofiber disruption, indistinct striations, poorly defined cellular boundaries, prominent inflammatory cell infiltration, and interstitial edema. Collectively, these findings indicate that coronary vascular tone alterations in septic patients perturb hemodynamics, while changes in cardiac index and ejection fraction variably compromise systolic and diastolic function, culminating in inadequate myocardial perfusion and ischemic hypoxia, which may ultimately precipitate SIC.

### 2.2 Cardiac myocyte death: apoptosis and autophagy

Autophagy is an intracellular process involving the self-degradation and recycling of long-lived proteins or organelles. When SIC occurs, the myocardium initiates autophagic mechanisms to minimize self-injury; therefore, maintaining the dynamic equilibrium between cardiomyocyte apoptosis and autophagy is essential during SIC progression to reduce adverse cardiac outcomes. Studies have demonstrated that autophagy exerts cytoprotective effects and enables adaptation to myocardial pathological states (13). Through this dynamic process, damaged cellular components, such as organelles and misfolded proteins, can be engulfed, packaged, and degraded via lysosomal fusion. When autophagy is suppressed in the heart, insufficient clearance of damaged mitochondria may further trigger apoptosis, thereby contributing to unfavorable cardiac outcomes in sepsis. Another *in vitro* study revealed that Periplaneta americana extract attenuated LPS-induced sepsis-associated myocardial injury by upregulating the expression of Mfn1, Mfn2, Opa1, and Drp1, thereby enhancing mitochondrial quality control through coordinated modulation of mitochondrial dynamics and mitophagy, ultimately reducing cardiomyocyte apoptosis (14). Shen et al. (15) investigated the effects of tumor necrosis factor-α (TNF-α) and interleukin-6 (IL-6) on Drp1 phosphorylation and distribution in cultured cells within an LPS-induced SIC model, finding that TNF-α might induce cardiomyocyte death through enhanced Drp1 levels and mitochondrial translocation. The interplay between inflammatory factors and cardiomyocyte death accelerates SIC progression; when cardiomyocyte apoptosis is perturbed, cardioprotective autophagy becomes suppressed, ultimately leading to irreversible myocardial injury and ischemic hypoxia.

### 2.3 Calcium dyshomeostasis

During the early phase of sepsis, intracellular calcium concentration in cardiomyocytes markedly increases, resulting in enhanced myocardial contractility. Excessive extracellular calcium influx exceeds the regulatory capacity of the sarcoplasmic reticulum (SR) and troponin, leading to intracellular calcium overload. Hobai et al. (16) proposed that calcium dyshomeostasis-induced SIC may be associated with the following factors: reduced L-type calcium channels, inhibition of SR calcium ATPase, ryanodine receptor abnormalities, and decreased myofilament calcium sensitivity. Cytosolic calcium overload constitutes a critical cause of cell death, as it activates a cascade of intracellular enzymatic reactions, inducing degradation of cytosolic proteins, plasma membrane, and nuclear DNA. Furthermore, Smeding et al. (17) observed scattered foci of actin-myosin contractile structure disruption in myocardial tissue from septic patients, which may contribute to abnormalities in excitation-contraction coupling. Phospholamban (PLN), a SR membrane protein, mediates calcium transport between the cytosol and SR; dephosphorylation of PLN enhances calcium pump activity and calcium flux, promoting myocardial contraction, whereas phosphorylation of PLN inhibits calcium pump activity and reduces calcium flux, facilitating myocardial relaxation. Collectively, these findings indicate that reduced L-type calcium channels and altered myofibrillar calcium sensitivity in sepsis induce significant disturbances in cardiomyocyte calcium homeostasis.

### 2.4 Inflammation-Mediated mechanisms

Excessive activation of inflammatory cells with massive release of inflammatory mediators constitutes one of the pivotal mechanisms in sepsis pathogenesis, while cytokines, including IL-1, IL-6, and IL-8 contribute to myocardial injury. An animal experiment demonstrated that TNF-α might serve as a critical inducer of Drp1 translocation during inflammatory injury of cardiomyocytes, potentially through enhancing p-Drp1 Ser616 levels and promoting mitochondrial translocation, thereby inducing cardiomyocyte death (15). In sepsis, abundant intracellular multiprotein complexes are extensively activated, such as the NOD-like receptor family pyrin domain-containing 3 (NLRP3) inflammasome, leading to increased maturation and release of IL-1β and IL-18, a reduction in intracellular cyclic AMP levels, and consequent suppression of myocardial contractility, ultimately resulting in cardiac dysfunction (18). Furthermore, immunoglobulin E (IgE) represents an independent biomarker of cardiovascular disease, with IgE-mediated inflammatory responses potentially causing platelet activation and arterial vasospasm. Current findings indicate that serum IgE-induced inflammatory immune responses in various tissues and organs represent not only type I hypersensitivity reactions but also serve as an independent indicator of SIC-associated immunosuppression following cardiac injury (19). Another study revealed increased neutrophil infiltration in the heart, liver, and lungs of infected animals, accompanied by decreased superoxide dismutase (SOD) and catalase (CAT) activities; conversely, dimethyl fumarate-treated infected animals exhibited markedly reduced neutrophil infiltration, elevated SOD and CAT activities following oxidative damage, and attenuated inflammatory injury in multiple organs (20). Collectively, these findings suggest that uncontrolled inflammatory cytokine release may represent the fundamental characteristic of severe trauma and sepsis. Appropriate modulation of excessive inflammatory responses may facilitate the control of SIC progression and holds promising therapeutic potential.

### 2.5 Immunomodulatory mechanisms

**Figure 1:**
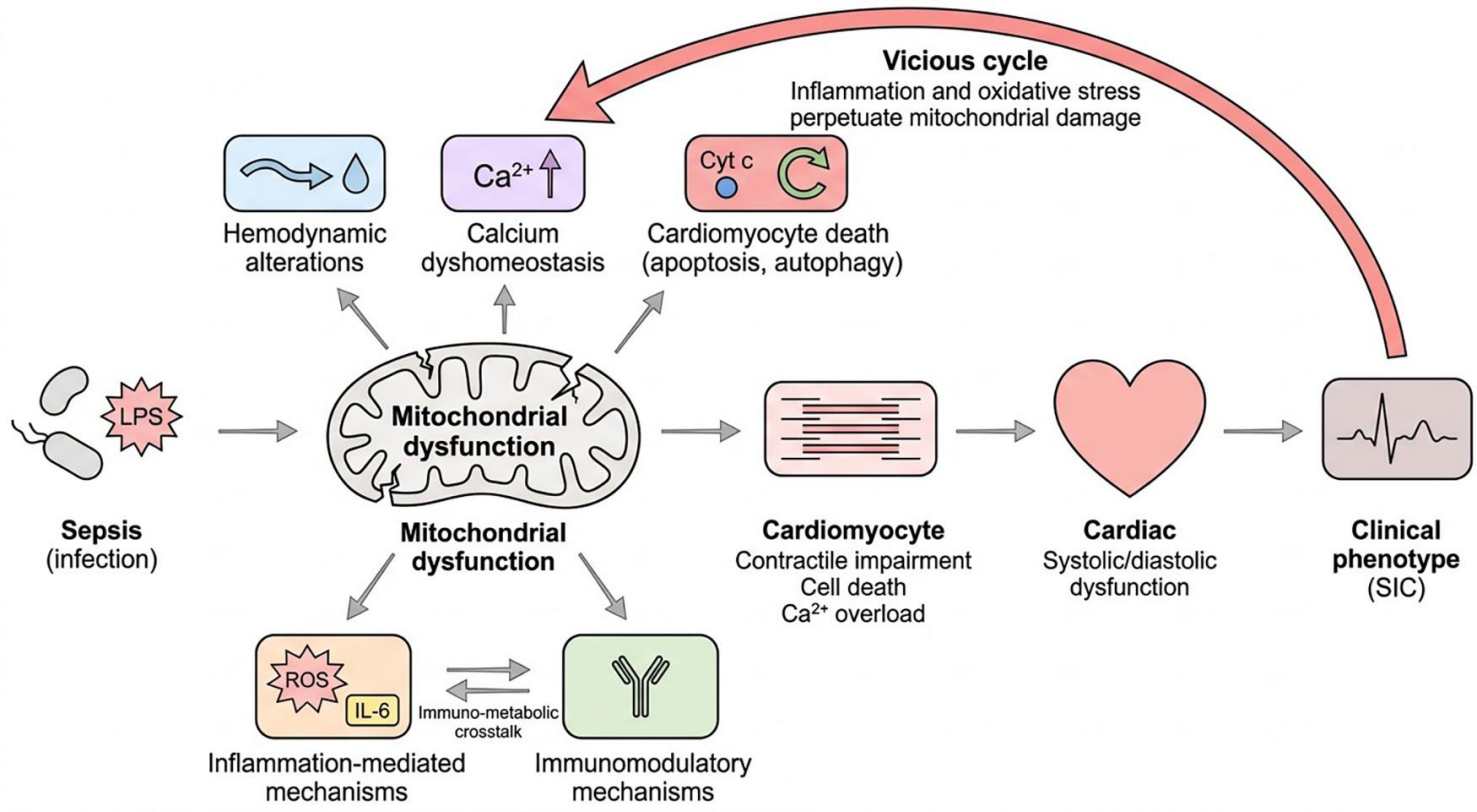
Conceptual framework integrating the pathophysiological mechanisms of sepsis-induced cardiomyopathy (SIC). Sepsis (infection) triggers lipopolysaccharide (LPS) release, which drives mitochondrial dysfunction as the central pathogenic hub. This mitochondrial pathology integrates five major pathophysiological processes that collectively propagate cardiac injury: hemodynamic alterations, calcium dyshomeostasis, cardiomyocyte death, inflammation-mediated mechanisms, and immunomodulatory mechanisms. Sepsis-induced peripheral vasodilation, decreased coronary artery compliance, and reduced ventricular elastance lead to diminished contractility and inadequate myocardial perfusion. Intracellular Ca² concentration markedly increases, exceeding sarcoplasmic reticulum regulatory capacity and causing cytosolic calcium overload. Apoptosis and autophagy dysregulation occurs, with cytochrome c (Cyt c) release from damaged mitochondria indicating mitochondrial outer membrane permeabilization (MOMP). Excessive reactive oxygen species (ROS) and interleukin-6 (IL-6) release establish immuno-metabolic crosstalk. Pattern recognition receptor signaling amplifies inflammatory responses. These processes converge to cause cardiomyocyte contractile impairment, cell death, and Ca² overload, which subsequently drive cardiac systolic/diastolic dysfunction and ultimately manifest as the clinical phenotype of SIC. A vicious cycle (indicated by the red curved arrow) is established whereby inflammation and oxidative stress perpetuate mitochondrial damage, further exacerbating disease progression.

Studies have shown that patients who died from sepsis exhibited marked immune paralysis (21). Therefore, how to enhance immunity in SIC patients at the stage of immune paralysis has become a current research hotspot. An animal experiment demonstrated that Toll-like receptor 4 (TLR4) and nuclear factor-kappa B (NF-*κ*B) protein expression were significantly enhanced in the LPS group, indicating that LPS-induced myocardial injury in rats was not only caused by activated immune cells but also by the activation of TLR4 on the cardiomyocyte surface, leading to NF-*κ*B activation and its subsequent nuclear translocation (22). This resulted in the expression of various mediators, including TNF-α, IL-6, IL-8, granulocyte-macrophage colony-stimulating factor, interferon-*β*, chemokines, and inducible nitric oxide synthase (iNOS), thereby participating in and amplifying the inflammatory response, ultimately exacerbating myocardial tissue injury. Ouyang et al. (23) demonstrated that MyD88 promoted neutrophil and macrophage recruitment, endothelial cell adhesion, and blood flow obstruction, consequently leading to myocardial injury; conversely, mice in the anti-MyD88 group maintained cardiac function with reduced cytokine release, decreased neutrophil infiltration, and attenuated apoptosis. These findings suggest that MyD88 inhibitors may protect the heart from severe sepsis-induced damage by suppressing cytokine release, reducing neutrophil infiltration, and alleviating cellular apoptosis. Collectively, these findings establish that TLR4/MyD88-mediated inflammatory signaling constitutes a central driver of SIC pathogenesis, with NF-*κ*B activation, cytokine amplification, and neutrophil-mediated tissue injury perpetuating cardiac dysfunction throughout disease progression. These inflammatory cascades converge upon and are amplified by mitochondrial dysfunction, which serves as the principal executor of cellular injury in SIC. An integrated conceptual framework linking these pathological processes is presented in Figure 1.

## 3 Mitochondrial pathology in SIC

Mitochondrial dysfunction in SIC follows a characteristic temporal sequence that begins with acute bioenergetic failure and progresses through oxidative stress amplification, inflammatory cascade activation, and ultimately quality control system collapse. Understanding this chronological hierarchy is essential for identifying early diagnostic biomarkers and prioritizing stage-specific therapeutic interventions.

### 3.1 Temporal sequence of mitochondrial dysfunction in SIC

The earliest detectable mitochondrial alteration in SIC is bioenergetic failure, occurring within hours of septic insult. Clinical studies demonstrate that septic shock patients exhibit significantly reduced myocardial mitochondrial ATP concentrations that correlate with shock severity and prognosis (24). At the molecular level, inflammatory mediators, including TNF-α and IL-1β, directly impair ETC complexes I and III, while iNOS-derived nitric oxide (NO) reacts with superoxide to form peroxynitrite, nitrating ETC components and collapsing membrane potential (25). This initial bioenergetic deficit represents the foundational event upon which subsequent pathological cascades build.

Following bioenergetic failure, typically within 6–12 h, mitochondrial reactive oxygen species (mtROS) production escalates dramatically. Impaired ETC function increases electron leak at complexes I and III, overwhelming antioxidant defenses and triggering oxidative stress (26, 27). ROS-mediated damage to mitochondrial DNA (mtDNA), lipids, and proteins creates a self-amplifying cycle: damaged mitochondria produce more ROS, which causes further mitochondrial damage. Notably, ROS-induced oxidation of ryanodine receptor 2 and sarcoplasmic/endoplasmic reticulum Ca^2+^-ATPase disrupts calcium handling, contributing to impaired excitation-contraction coupling (28).

The third phase, emerging within 12–24 h, involves mitochondrial DAMP release and inflammasome activation. As mitochondrial membranes become compromised, mtDNA, cardiolipin, and ATP escape into the cytoplasm, activating the cGAS-STING, TLR9, and NLRP3 pathways (29–31). These events transform mitochondria from passive victims of inflammation into active drivers of the innate immune response. The NLRP3 inflammasome activation is particularly critical, as it drives IL-1β and IL-18 maturation, which further suppress myocardial contractility and perpetuate mitochondrial damage (32).

In the later stages (24–72 h), mitochondrial quality control systems begin to fail. Mitophagy, initially enhanced as a protective response, becomes impaired due to Parkin degradation by caspase-1 and inflammatory cytokine-mediated suppression of the PINK1-Parkin pathway (33). Simultaneously, mitochondrial dynamics shift toward excessive fission mediated by the Drp1/Fis1 interaction, while fusion proteins Mfn1/2 and OPA1 are downregulated, resulting in mitochondrial network fragmentation (34, 35). These changes prevent clearance of damaged mitochondria and exacerbate the accumulation of dysfunctional organelles.

The final phase involves intercellular mitochondrial transfer through TNTs, which exhibits paradoxical dual effects. Initially, mitochondria serve as compensatory inputs to maintain cellular metabolism; however, as disease progresses, the transfer direction reverses to export damaged mitochondria from cardiomyocytes to surrounding cells, becoming a critical event driving continuous cardiac deterioration (36).

### 3.2 Priority therapeutic targets based on temporal hierarchy

Given this temporal sequence, therapeutic interventions should be stratified according to disease stage. Early-stage targets (0–6 h) should prioritize preservation of bioenergetic function. Mitochondria-targeted antioxidants such as MitoQ and SS-31, which scavenge mtROS and maintain membrane potential, represent the most promising candidates for this window (10, 11, 37). SS-31 additionally stabilizes cardiolipin and inhibits NF-*κ*B signaling, offering dual protective mechanisms (11).

For the intermediate phase (6–24 h), suppressing DAMPs-mediated inflammation becomes critical. Inhibitors of the cGAS-STING pathway, TLR9 antagonists, and NLRP3 inflammasome inhibitors such as MCC950 have demonstrated cardioprotective effects in preclinical models by blocking the transition from mitochondrial damage to innate immune activation (38, 39). The caspase-1 inhibitor VX765, by preventing Parkin degradation, preserves mitophagy capacity and attenuates inflammatory injury (40).

In late-stage SIC (24–72 h), restoring mitochondrial quality control systems offers therapeutic potential. Enhancing mitophagy through Tat-Beclin-1 peptide or Po-Ge-Jiu-Xin Decoction-mediated PINK1-Parkin pathway activation can clear accumulated damaged mitochondria (41, 42). Modulating mitochondrial dynamics to restore the fission-fusion balance, such as inhibiting Drp1 translocation or promoting Mfn2 expression, may rescue mitochondrial network integrity (34, 35).

Melatonin represents a unique multi-stage therapeutic candidate. By inhibiting Ripk3, melatonin improves mitochondrial bioenergetics, reduces ROS, sustains mitochondrial dynamics, and ameliorates ER stress simultaneously, thereby targeting multiple phases of the pathological cascade (43).

However, the clinical translation of these strategies faces substantial challenges. The narrow therapeutic window for early intervention, the heterogeneity of human sepsis, and the lack of real-time mitochondrial function monitoring at the bedside complicate stage-specific therapy implementation (44–46). Future research should prioritize the development of biomarkers capable of distinguishing these temporal phases in individual patients, enabling precision medicine approaches to mitochondria-targeted therapy in SIC.

### 3.3 Mitochondria as central regulators of inflammation

SIC is a persistent inflammatory response. Mitochondria play an important role in controlling inflammatory responses. In addition to serving as ligands for pattern recognition receptors (PRRs) due to their similarity to bacterial components, thereby triggering immune responses, the double-membrane structure prevents abnormal release of mitochondrial DAMPs that would aberrantly activate PRR signaling pathways. Furthermore, mitochondria can control apoptosis and necrosis. MOMP is the core link in mitochondrial apoptosis and immune response participation. MOMP is activated by inflammatory factors such as LPS and TNF-α, as well as ROS. Through the Bcl-2 family proteins Bax/Bak, oligomeric pores are formed. These pores release pro-apoptotic factors from the mitochondrial intermembrane space, such as cytochrome c (Cyt C). These activate the endogenous apoptotic pathway through caspase cascade reactions. Moreover, intramitochondrial mtDNA, cardiolipin, and ATP are released into the cytoplasm. These substances serve as mitochondrial DAMPs recognized by cytoplasmic PRRs, thereby activating innate immunity (9). As one of the main components of mitochondrial DAMPs, mtDNA triggers immune responses primarily through two signaling pathways.

#### 3.3.1 mtDNA-cGAS-STING signaling in SIC progression

Escaped mtDNA binds cGAS, catalyzing cGAMP production. cGAMP activates STING, which recruits TANK-binding kinase 1 to phosphorylate interferon regulatory factor 3, driving type I interferon and NF-*κ*B expression and upregulating TNF-α and IL-6 (38). In SIC, iNOS exacerbates pressure overload-induced cardiac dysfunction through cytoplasmic mtDNA-mediated cGAS-STING activation. iNOS knockout eliminates mtDNA release, reduces cardiac inflammation, and improves cardiac function, whereas cGAS-STING activation abrogates these protective effects (39). These findings establish the mtDNA-cGAS-STING pathway as a critical therapeutic target for interrupting SIC progression.

#### 3.3.2 mtDNA-TLR9 axis in SIC cardiac injury

mtDNA contains unmethylated CpG motifs structurally similar to bacterial DNA, which TLR9 recognizes. TLR9 activation initiates MyD88-dependent downstream signaling, activating the NF-*κ*B and MAPK pathways to drive massive transcription and release of pro-inflammatory cytokines, amplifying the inflammatory response (47). While the mtDNA-TLR9 pathway has been primarily characterized in pulmonary vascular injury, emerging evidence indicates its direct contribution to SIC. In septic cardiomyopathy, TLR9 activation on cardiac macrophages and fibroblasts propagates NF-*κ*B-driven IL-6 and TNF-α release, exacerbating myocardial inflammatory infiltration and contractile dysfunction. MyD88 inhibition attenuates neutrophil recruitment and preserves cardiac function in septic models (29), suggesting that interrupting mtDNA-TLR9 signaling may limit cardiac-specific inflammatory damage in SIC.

#### 3.3.3 NLRP3 inflammasome activation in SIC pathogenesis

**Figure 2:**
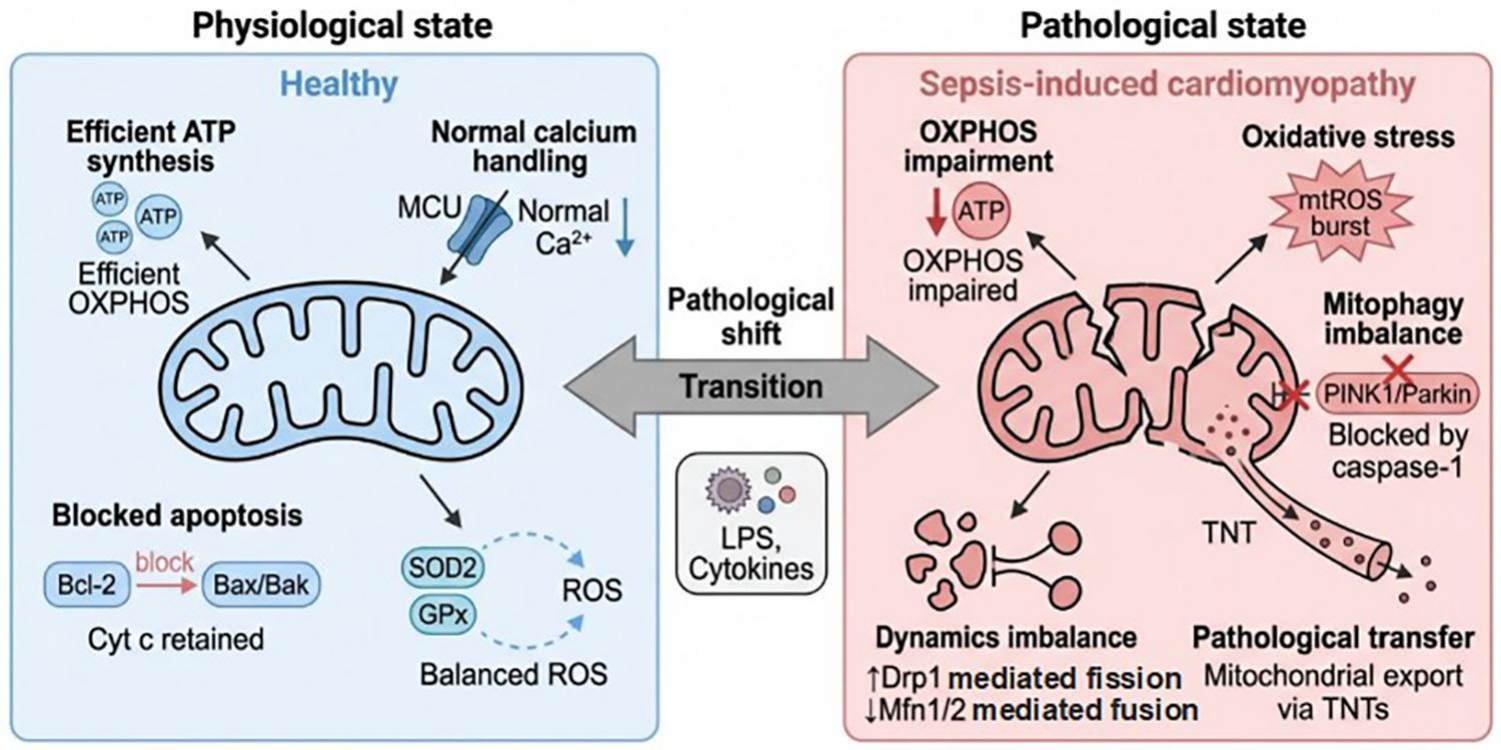
Comparison of mitochondrial features under physiological and pathological states in sepsis-induced cardiomyopathy (SIC). (Left panel: Physiological state) Under healthy conditions, cardiomyocyte mitochondria maintain homeostasis through four key features: (1) Efficient ATP synthesis via intact oxidative phosphorylation (OXPHOS) and normal electron transport chain function; (2) Normal calcium handling through the mitochondrial calcium uniporter (MCU), ensuring balanced Ca^2+^ flux; (3) Blocked apoptosis via Bcl-2-mediated inhibition of Bax/Bak oligomerization, with cytochrome c (Cyt c) retained within the mitochondrial intermembrane space; and (4) Balanced ROS production counteracted by antioxidant enzymes including superoxide dismutase 2 (SOD2) and glutathione peroxidase (GPx). (Right panel: Pathological state) During sepsis-induced cardiomyopathy, LPS and inflammatory cytokines drive a pathological shift characterized by: (1) OXPHOS impairment with reduced ATP synthesis due to electron transport chain dysfunction; (2) Oxidative stress burst with excessive mitochondrial reactive oxygen species (mtROS) production overwhelming antioxidant defenses; (3) Mitophagy imbalance with the PINK1/Parkin pathway blocked by caspase-1-mediated Parkin degradation, preventing clearance of damaged mitochondria; (4) Dynamics imbalance featuring excessive Drp1-mediated mitochondrial fission and suppressed Mfn1/2-mediated fusion, resulting in mitochondrial network fragmentation; and (5) Pathological transfer of damaged mitochondria via tunneling nanotubes (TNTs) that propagate cellular injury to neighboring cardiomyocytes.

The mtDNA-NLRP3 axis plays a central role in SIC. SIC causes mitochondrial structural and functional damage, increasing mtROS and mtDNA release, which promote NLRP3 oligomerization and exacerbate myocardial injury (30). As a DAMP, mtDNA co-immunoprecipitates with NLRP3, specifically activating the inflammasome. When mitochondria are destabilized, cardiolipin redistributes to the outer mitochondrial surface and directly binds the LRR domain of NLRP3 to activate it (31). Mitochondria not only directly activate NLRP3 by releasing ROS, mtDNA, and cardiolipin, but also recruit NLRP3 and promote its assembly through mitochondrial antiviral-signaling protein and Mfn2 (31). GAS6 inhibits NLRP3 activation through the AXL receptor, improving sepsis-induced cardiac dysfunction, alleviating mitochondrial damage, ER stress, oxidative stress, and apoptosis; GAS6 knockout eliminates these protective effects (48). IP3R2-mediated ER Ca^2+^ release promotes LPS-induced cardiomyocyte pyroptosis through NLRP3/Caspase-1/GSDMD activation, which IP3R2 inhibition reverses (49). NLRP3 gene knockout preserves systolic and diastolic function in septic mice and reduces cardiac and cardiomyocyte atrophy. IL-1β causes cardiomyocyte atrophy and systolic/diastolic dysfunction by activating NF-*κ*B and its target genes (32). In SIC, the release of inflammatory factors resulting from these events further exacerbates mitochondrial damage and OMM permeabilization, creating a vicious cycle of inflammatory response. Excessive release of inflammatory cytokines leads to increased vascular permeability, organ hypoperfusion, and ultimately cardiac dysfunction. Collectively, these findings establish that mitochondrial DAMPs activate multiple inflammatory cascades (see Figure 2).

### 3.4 Mitochondrial energy metabolism dysfunction in SIC

#### 3.4.1 Mitochondrial OXPHOS impairment in SIC

Under steady-state conditions, cardiomyocytes predominantly rely on mitochondrial OXPHOS for ATP generation. During sepsis, iNOS activation produces NO, which rapidly reacts with superoxide to generate peroxynitrite, nitrating ETC components and collapsing membrane potential (25). Consequently, the transmembrane proton gradient dissipates and ATP generation markedly decreases. Additionally, SIC frequently accompanies microcirculatory perfusion disturbances and hypermetabolic demands. Cellular oxygen utilization becomes limited. Tissue metabolism rapidly switches from OXPHOS toward aerobic glycolysis. Although this metabolic reprogramming transiently supports cell survival and pro-inflammatory responses, prolonged maintenance leads to energy depletion, immunometabolic imbalance, and multiple organ failure due to ATP reduction, lactate accumulation, and redox imbalance.

Recent investigations have further elucidated the complex mechanisms underlying energy metabolic disturbances in SIC. LPS-induced SIC is associated with profound suppression of energy substrate catabolism. Oxidation of fatty acids, glucose, and ketone bodies all became inhibited. Simultaneously, myocardial glycogen and triglyceride accumulated. This “metabolic stagnation” state is closely related to mitochondrial dysfunction. The fuel shifted from fatty acids toward glucose/ketone bodies might prove detrimental rather than protective under septic conditions (50). Clinical investigations demonstrated that septic shock patients exhibited significantly reduced myocardial mitochondrial ATP concentrations. These levels correlated with shock severity and prognosis. Mitochondrial complex I activity inversely related to norepinephrine requirements. It positively correlated with ATP concentrations. These findings implicated bioenergetic failure as an important pathophysiological mechanism underlying multiple organ dysfunction (24). Critically ill septic patients with multiple organ failure showed reduced mitochondrial content in both intercostal and leg muscles. Energy-rich phosphate concentrations decreased. Anaerobic energy production increased in leg muscles. These observations further confirmed systemic metabolic disturbances in peripheral tissues (51).

At the molecular mechanism level, septic hearts display widespread down-regulation of mitochondrial and contractile protein gene expression. Compared with patients having ischemic heart disease or dilated cardiomyopathy, septic patients showed a 43% reduction in mRNA levels of 198 mitochondrially localized energy production genes, including TCA cycle and electron transport genes. mRNA levels of 9 genes responsible for sarcomere contraction and excitation-contraction coupling also decreased by 43%. This global down-regulation of gene expression might represent a short-term adaptive response of the heart to acute cardiovascular homeostatic changes (52). Peroxisome proliferator-activated receptor *γ* (PPAR*γ*) activation prevented sepsis-related cardiac dysfunction and mortality. This protection occurred despite persistent cardiac inflammation and PPAR*α* down-regulation. These findings suggested that maintaining fatty acid oxidation through activation of alternative nuclear receptors may constitute a therapeutic strategy (53).

Clinical observations indicate that reversible myocardial depression in human septic shock does not result from global ischemia. Patients developing myocardial suppression demonstrated normal or even increased coronary blood flow. No net myocardial lactate production occurred. The oxygen content difference narrowed. Arterial oxygen fractional extraction decreased. This pattern of disordered coronary autoregulation resembled arteriovenous shunting observed in other organs (54). Intramyocardial lipid deposition also represented a metabolic characteristic of non-ischemic heart failure. This phenomenon appeared particularly prominent in diabetic and obese patients. It is associated with up-regulation of PPAR*α*-regulated genes, *β*-myosin heavy chain, and TNF-α. These findings suggest the involvement of lipotoxicity in myocardial dysfunction (55).

#### 3.4.2 Immune cell metabolic reprogramming in SIC

Macrophage metabolic status is precisely regulated by metabolic reprogramming during SIC. In early sepsis, LPS and IFN-*γ* stabilize HIF-1*α* and activate mTOR, driving M1 polarization and glycolytic metabolism. This metabolic shift inhibits mitochondrial function through the iNOS-NO axis, locking cells in a pro-inflammatory state. In late sepsis, persistent damage impairs both glycolysis and OXPHOS, leading to energy exhaustion and immune paralysis (56).

Multiple regulatory molecules modulate this process. ACE2 alleviates oxidative stress and M1 polarization through the Ang(1–7)/MasR axis (57). ZBP1 drives M1 polarization via STAT1, while its deficiency promotes M2 conversion (58). ADAM8 impairs efferocytosis, exacerbating cardiac injury (59). Notch1 suppresses mitophagy through Mst1 upregulation, increasing ROS and inflammation (60). PPAR*α* enhances M2 polarization via DUSP1 promoter binding (61). Metabolically, glycolytic enzymes and TCA intermediates promote NLRP3 activation, whereas itaconate exerts inhibitory effects (62).

### 3.5 Mitochondrial oxidative stress in SIC

Oxidative stress denotes the imbalance between ROS generation and antioxidant defense systems within mitochondria. Excessive ROS induces oxidative damage to mitochondrial proteins, membranes, and mtDNA. This ultimately culminates in irreversible cellular injury and apoptosis (26). During sepsis, inflammatory mediators such as NO induced by LPS suppress ETC complexes I, III, and IV. This aggravates electron leakage and drives massive production of mtROS. Consequently, oxidative stress is triggered. The principal pathogenic mechanisms encompass the following. High concentrations of mtROS provoke opening of the mitochondrial permeability transition pore (mPTP). This results in the release of apoptotic factors, including Cyt C, depletion of ATP, and activation of caspase cascade reactions. These events directly initiate apoptotic cell death. Oxidized mtDNA functions as DAMP molecules. It not only activates the NLRP3 inflammasome but also exacerbates mitochondrial damage through downstream signals such as caspase-1. A vicious cycle thus forms (63). Furthermore, ROS can activate pivotal inflammatory pathways and amplify inflammatory responses. Studies have demonstrated that during oxidative stress, inhibitor of NF-*κ*B kinase promotes phosphorylation and degradation of inhibitor of NF-*κ*B. This activates NF-*κ*B. Activated NF-*κ*B subsequently translocates into the nucleus and initiates transcription of pro-inflammatory factors, including TNF-α and IL-6 (64). Additional studies have shown that ROS can also activate MAPK family members such as extracellular signal-regulated kinase (ERK), c-Jun N-terminal kinase (JNK), and p38 MAPK. These kinases regulate cellular inflammatory responses (65). Oxidative stress plays a central role in the pathogenesis of SIC. Under septic conditions, mitochondrial dysfunction causes massive superoxide anion release from the ETC. Simultaneously, the nicotinamide adenine dinucleotide phosphate oxidase (NOX) system becomes excessively activated. Together, these produce explosive accumulation of ROS. This oxidative stress milieu directly damages myocardial cell membrane lipids, proteins, and DNA. It triggers inflammatory cascade reactions and eventually leads to myocardial contractile dysfunction and diminished cardiac pumping capacity (28). Therefore, targeting ROS regulatory pathways for antioxidant intervention has emerged as a major therapeutic direction for SIC.

### 3.6 The role of mitophagy in SIC

#### 3.6.1 Mechanisms of mitophagy

The underlying mechanisms involve two distinct pathways: the PINK1-Parkin-dependent pathway, mediated by the coordinated action of the serine/threonine kinase PINK1 and the E3 ubiquitin ligase Parkin, and the PINK1-Parkin-independent pathway. Upon mitochondrial damage, the loss of mitochondrial membrane potential stabilizes PINK1 on the OMM, where it undergoes autophosphorylation and activation, subsequently recruiting and activating Parkin. Activated Parkin then ubiquitinates OMM proteins, and these ubiquitin signals further recruit additional Parkin, establishing a positive feedback loop that amplifies the mitochondrial damage signal. Ultimately, ubiquitinated OMM proteins are recognized by autophagy adaptors containing LC3-interacting regions (LIR), such as p62, optineurin, and TAX1-binding protein 1, which mediate interaction with LC3 and facilitate the engulfment of damaged mitochondria by autophagosomes (66, 67). Additionally, certain OMM proteins, including BNIP3, can function as autophagy receptors that directly bind LC3 through their LIR domains to induce mitophagy in the absence of Parkin.

#### 3.6.2 The relationship between SIC and mitophagy

In the early stage of SIC, mitophagy is enhanced to clear damaged mitochondria, maintain cardiomyocyte homeostasis, and exert protective effects. However, as sepsis progresses, mitophagy function becomes impaired, leading to the accumulation of damaged mitochondria, increased ROS release, and exacerbation of myocardial dysfunction. LPS or inflammatory cytokines activate the NLRP3 inflammasome through the TLR4/MyD88 signaling pathway, resulting in caspase-1 activation and translocation to mitochondria. This process degrades the Parkin protein and disrupts the PINK1-Parkin pathway, thereby blocking mitophagy and aggravating myocardial injury (33). Furthermore, mtROS and mtDNA during SIC can directly activate NLRP3, further inhibiting this pathway (31).

Multiple pharmacological and genetic strategies have demonstrated efficacy in restoring mitophagy function during SIC progression. The caspase-1 inhibitor VX765 enhances macrophage mitophagy, promotes M2 polarization, and improves efferocytosis, thereby alleviating mitochondrial damage through STAT1/caspase pathway inhibition (40). The autophagy-related protein Beclin-1 reduces mitochondrial DAMPs release, activates the PINK1-Parkin pathway, inhibits mTOR signaling, and participates in positive feedback regulation of autophagy through enhanced AMPK (41). Prohibitin 1 (PHB1) negatively regulates NLRP3 inflammasome activation by modulating the LC3-mitophagy pathway interaction (68). In traditional Chinese medicine, Po-Ge-Jiu-Xin Decoction improves mitochondrial quality by activating PINK1/Parkin-mediated mitophagy (69). Additionally, FUNDC1-mediated mitophagy plays a crucial protective role; its deficiency leads to mitochondrial dysfunction and decreased contractility (70), while DUSP1 attenuates sepsis-induced myocardial injury by improving mitophagy and mitochondrial metabolism (71). Conversely, PTPN1 impairs mitophagy via STAT3 dephosphorylation, inhibiting the PINK1/Parkin pathway (72), and BNIP3 serves as a compensatory mechanism directly binding LC3 to induce mitophagy when Parkin is absent (33).

Overall, mitophagy is enhanced in the early stage of SIC but becomes suppressed at later stages. The decline in autophagic flux exacerbates myocardial injury. Monitoring LC3II and p62 can serve as markers for assessing myocardial autophagic flux (33). Therefore, selecting appropriate timing to intervene in autophagy represents a key therapeutic strategy for SIC.

### 3.7 Impact of mitochondrial dynamics imbalance on SIC

In SIC, the delicate balance is disrupted, characterized by excessive mitochondrial fission and suppressed fusion, leading to mitochondrial network fragmentation, energy metabolic dysfunction, and cardiomyocyte death (34).

During the early stage of SIC, LPS and inflammatory cytokines activate mitochondrial fission through multiple signaling pathways. Drp1, a large GTPase that serves as the core mediator of mitochondrial fission, translocates from the cytosol to the OMM upon LPS stimulation (34). At the OMM, Drp1 interacts with fission protein 1 (Fis1), forming ring-like multimeric structures that constrict and divide mitochondria, thereby driving pathological fission (34). The Drp1/Fis1 interaction has been identified as a predominant driver of mitochondrial dysfunction in SIC, with this pathway being specifically activated under septic conditions (34). Additionally, stress-responsive protein 2 (SRV2) promotes mitochondrial fission through the Mst1/Drp1 signaling axis, exacerbating oxidative damage, ATP depletion, and ultimately cardiomyocyte death (73). Mitochondria-associated ubiquitin ligase (MAPL) contributes to mitochondrial dynamics imbalance through dual mechanisms: facilitating Drp1 SUMOylation to enhance its protein stability and membrane binding capacity, while simultaneously promoting Mfn2 degradation via ubiquitination to suppress fusion (74). Furthermore, DNA-dependent protein kinase catalytic subunit (DNA-PKcs) disrupts inositol polyphosphate-1-phosphatase 2 (INF2)-dependent mitochondrial dynamics, thereby aggravating the fission-fusion imbalance (75).

Concomitantly, mitochondrial fusion is markedly suppressed in SIC. LPS stimulation downregulates the expression of fusion proteins, including mitofusin 1/2 (Mfn1/2) and OPA1, resulting in mitochondrial network fragmentation and compromised mtDNA stability and OXPHOS efficiency (35). This imbalance in mitochondrial dynamics not only alters mitochondrial morphology but also induces calcium homeostasis disturbance, excessive ROS production, and metabolic reprogramming, ultimately triggering cardiomyocyte apoptosis and necroptosis (69).

Moreover, mitochondrial dynamics imbalance exhibits complex crosstalk with mitophagy, calcium homeostasis, and energy metabolism. While excessive mitochondrial fission may facilitate the segregation and clearance of damaged mitochondria under physiological conditions, this process is compromised in SIC due to impaired autophagic flux, leading to the accumulation of dysfunctional mitochondria (34). Concurrently, fragmented mitochondrial networks disrupt the spatial coordination of calcium uptake and release, exacerbating calcium overload and further activating the opening of mPTP, thereby triggering Cyt C release and apoptotic cascade activation (34).

Collectively, mitochondrial dynamics imbalance represents a central pathogenic mechanism in the development and progression of SIC, manifested as Drp1/Fis1-mediated excessive fission and Mfn2/OPA1-dependent fusion impairment. These alterations collectively contribute to myocardial dysfunction through their detrimental effects on mitochondrial morphology, energy metabolism, calcium homeostasis, and cell death pathways.

### 3.8 Mitochondrial transfer in SIC

Mitochondrial transfer exhibits paradoxical dual effects in SIC, serving both as a mechanism of damage propagation and as an endogenous repair pathway. During the early stage of sepsis, LPS and inflammatory cytokines stimulate TNT formation between cardiomyocytes and cardiac resident cells, enabling intercellular transfer of damaged mitochondria that induces metabolic reprogramming, ROS accumulation, and inflammatory amplification in recipient cells, thereby exacerbating myocardial injury (36, 76). Conversely, mesenchymal stem cell (MSC)-derived mitochondria-enriched extracellular vesicles (M-EVs) promote myocardial ATP generation through PGC-1*α* activation, enhance antioxidant function, and attenuate inflammatory responses, conferring significant cardioprotection in sepsis models (36, 77).

The directionality of mitochondrial transfer undergoes dynamic changes during SIC progression. Initially, mitochondria serve as compensatory inputs to maintain cellular metabolism; however, as disease progresses, the transfer direction reverses to export damaged mitochondria from cardiomyocytes to surrounding cells, driving continuous cardiac deterioration (36, 78). This “communication direction reversal” suggests that mitochondria transform from passive victims into upstream pathogenic factors through altered intercellular communication (36). The sirtuin 1 (SIRT1)/PGC-1*α* axis, a key regulator of mitochondrial biogenesis and dynamics, is reactivated following therapeutic mitochondrial transfer, restoring mitochondrial function and attenuating myocardial injury (36).

Beyond TNT-mediated transfer, platelet-derived mitochondrial release contributes to systemic inflammatory amplification in sepsis through activation of secreted phospholipase A2 (79). Additionally, astrocyte-to-neuron mitochondrial transfer has been documented in ischemic brain injury, establishing a broader paradigm of intercellular mitochondrial exchange in critical illness (78). These collective findings indicate that mitochondrial transfer represents a complex, context-dependent process in SIC, with therapeutic strategies requiring precise modulation of transfer directionality to promote repair while preventing pathological propagation (80).

**Figure 3:**
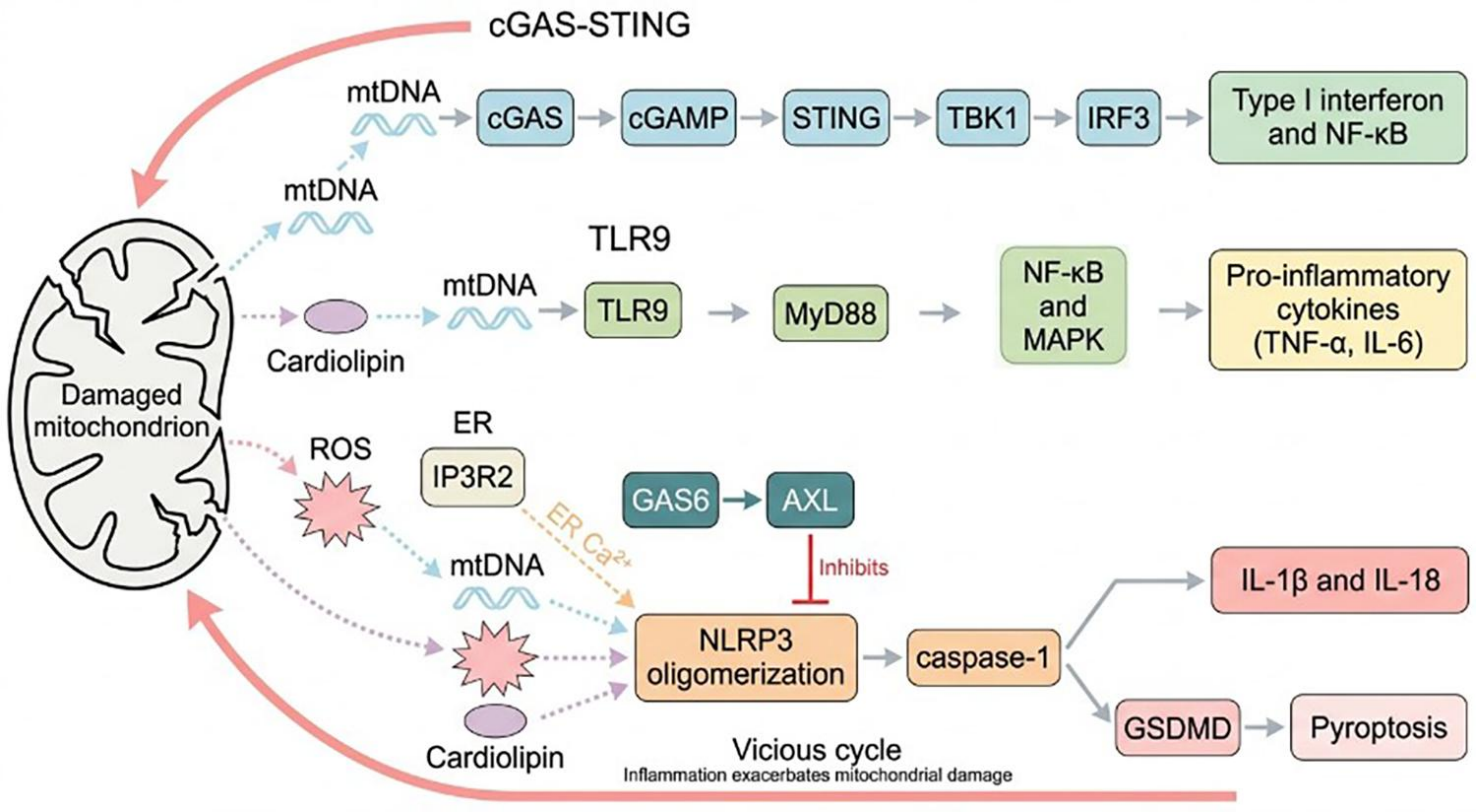
Mitochondrial DAMPs activate multiple inflammatory cascades in sepsis-induced cardiomyopathy (SIC). Damaged mitochondria release mitochondrial DNA (mtDNA), cardiolipin, and reactive oxygen species (ROS) into the cytoplasm, activating three parallel innate immune signaling pathways that converge to amplify cardiac inflammation and establish a vicious cycle of mitochondrial damage. Escaped mtDNA binds cyclic GMP-AMP synthase (cGAS), catalyzing cGAMP production; cGAMP activates stimulator of interferon genes (STING), which recruits TANK-binding kinase 1 (TBK1) to phosphorylate interferon regulatory factor 3 (IRF3), driving type I interferon and NF-κB expression. mtDNA containing unmethylated CpG motifs is recognized by Toll-like receptor 9 (TLR9), initiating MyD88-dependent downstream signaling that activates NF-κB and MAPK pathways to drive massive transcription and release of pro-inflammatory cytokines (TNF-α, IL-6). mtDNA, ROS, and cardiolipin from damaged mitochondria, together with endoplasmic reticulum (ER) Ca² release via inositol 1,4,5-trisphosphate receptor type 2 (IP3R2), converge to promote NLRP3 oligomerization. This leads to caspase-1 activation and subsequent interleukin-1β (IL-1β) and interleukin-18 (IL-18) release, as well as gasdermin D (GSDMD)-mediated pyroptosis. The GAS6/AXL axis negatively regulates NLRP3 activation, providing an endogenous inhibitory mechanism. The red curved arrow indicates that inflammation exacerbates mitochondrial damage, perpetuating the pathological cycle.

The key molecular regulators involved in mitochondrial dynamics imbalance, mitophagy impairment, and mitochondrial transfer are summarized in Table 1. Collectively, the major differences between physiological mitochondrial function and the pathological alterations in SIC are summarized in Figure 3.

**Table 1:** Key molecular regulators of mitochondrial dynamics, mitophagy, and intercellular transfer in SIC.

Regulator	Type/function	Effect in SIC	Mechanism	References
Drp1	Fission promoter	Excessive fission, mitochondrial fragmentation	Translocates to the OMM, interacts with Fis1, and forms ring-like structures	(34)
Fis1	Fission adaptor	Promotes pathological fission	Recruits Drp1 to the mitochondrial surface	(34)
Mfn1/2	Fusion mediator	Downregulated, suppressed fusion	LPS reduces expression, leading to network fragmentation	(35)
OPA1	Fusion/cristae regulator	Decreased expression	Impairs inner membrane fusion and mtDNA stability	(35)
SRV2	Fission regulator	Promotes fission via the Mst1/Drp1 axis	Exacerbates oxidative damage and ATP depletion	(73)
MAPL	Dual regulator	Drp1 SUMOylation, Mfn2 degradation	Dual mechanisms exacerbate dynamics imbalance	(74)
DNA-PKcs	Kinase	Disrupts INF2-dependent dynamics	Aggravates fission-fusion imbalance	(75)
PINK1	Mitophagy initiator	Blocked by caspase-1 in late sepsis	Stabilizes on the OMM, recruits Parkin	(33)
Parkin	E3 ubiquitin ligase	Degraded by caspase-1, impaired mitophagy	Ubiquitinates OMM proteins, recruits autophagic adaptors	(33)
FUNDC1	Mitophagy receptor	Protective; deficiency worsens dysfunction	Protective; deficiency worsens dysfunction	(70)
BNIP3	Mitophagy receptor	Serves as backup when PINK1-Parkin is blocked	Directly binds LC3, inducing mitophagy	(33)
PHB1	Mitophagy promoter	Negatively regulates the NLRP3 inflammasome	Modulates LC3 interaction, inhibits inflammation	(68)
DUSP1	Inflammatory regulator	Improves mitophagy and mitochondrial metabolism	Attenuates sepsis-induced myocardial injury	(71)
PTPN1	Phosphatase	Impairs mitophagy via STAT3 dephosphorylation	Inhibits the PINK1/Parkin pathway	(72)
TNTs	Intercellular structure	Mediates pathological mitochondrial export	Drp1 interacts with Filamin/Kinesin, drives TNT formation	(36)
MSC-EVs	Extracellular vesicle	Protective mitochondrial transfer	Promotes ATP generation via PGC-1*α*, and reduces oxidative stress	(36)

Drp1, Dynamin-related protein 1; Fis1, Fission 1; Mfn1/2, Mitofusin 1/2; OPA1, Optic atrophy 1; SRV2, Stress-responsive protein 2; MAPL, Mitochondria-anchored protein ligase; DNA-PKcs, DNA-dependent protein kinase catalytic subunit; PINK1, PTEN-induced putative kinase 1; Parkin, PARK2 E3 ubiquitin-protein ligase; FUNDC1, FUN14 domain containing 1; BNIP3, BCL2/adenovirus E1B 19 kDa protein-interacting protein 3; PHB1, Prohibitin 1; DUSP1, Dual specificity phosphatase 1; PTPN1, Protein tyrosine phosphatase non-receptor type 1; TNTs, Tunneling nanotubes; MSC-EVs, Mesenchymal stem cell-derived extracellular vesicles; OMM, Outer mitochondrial membrane; mtDNA, Mitochondrial DNA; LPS, Lipopolysaccharide; Mst1, Mammalian sterile 20-like kinase 1; INF2, Inverted formin 2; LC3, Microtubule-associated protein 1 light chain 3; NLRP3, NOD-like receptor family pyrin domain containing 3; STAT3, Signal transducer and activator of transcription 3; PGC-1α, Peroxisome proliferator-activated receptor gamma coactivator 1-alpha; SIC, Sepsis-induced cardiomyopathy.

## 4 Treatment strategies: targeting mitochondria

Mitochondrial-targeted therapeutic strategies for SIC can be systematically categorized into five major classes: (1) mitochondria-targeted antioxidants that scavenge ROS and preserve redox homeostasis; (2) mitochondrial dynamics regulators that restore fission-fusion balance; (3) mitochondrial biogenesis activators that promote mitochondrial renewal; (4) mitophagy modulators that enhance quality control; and (5) inflammation-mitochondria axis modulators that interrupt the vicious cycle between mitochondrial damage and inflammatory amplification. The major mitochondria-targeted therapeutic strategies for SIC are summarized in Table 2.

**Table 2:** Systematic classification of mitochondria-targeted therapeutic strategies for SIC.

Intervention strategies	Types		Models	Mechanisms	References
Mitochondria-targeted antioxidants	Lipophilic cation-conjugated antioxidants	MitoQ	LPS-induced sepsis rat/mouse model	MitoQ selectively accumulates in mitochondria via the TPP⁺ cation, scavenges mtROS, maintains mitochondrial membrane potential and respiration, preserves ATP generation and cardiac pressure, inhibits caspase activation, and suppresses pro-inflammatory cytokines.	(10, 37)
		Mito-Vit-E	Pneumonia-related sepsis rat model	Mito-Vit-E protects mtDNA integrity, blocks TLR9/NF-κB signaling, suppresses oxidative stress and IL-1β, reduces cardiac injury markers, and improves cardiac function and survival.	(81, 82)
		SOD mimetics: Tempol; EUK-8	CLP sepsis rat model; Endotoxic shock rat model	Tempol upregulates Klotho, inhibits p38-MAPK, reduces TNF-α and caspase-3, restores SOD and glutathione, and attenuates cardiorenal injury; EUK-8 dose-dependently attenuates organ injury and delays hypotension through dual SOD/CAT activity.	(83, 84)
		Vitamin C	LPS-induced sepsis rat model; Freshly isolated adult rat cardiomyocytes exposed to singlet oxygen	Vitamin C pretreatment reduces oxidative stress, restores SOD activity, decreases TNF-α and IL-6, and protects cardiomyocyte structure; combined with vitamin E, it produces synergistic protection through regeneration of α-tocopherol.	(85, 86)
		Combination therapeutic strategies	94 patients with septic shock	The “metabolic resuscitation” triple therapy did not significantly improve mortality, renal replacement therapy requirement, length of stay, or vasopressor independence compared to standard care.	(87)
	Peptide-based mitochondrial antioxidants	SS-31	LPS-induced sepsis mouse model; Doxorubicin-induced cardiotoxicity mouse model; CLP sepsis mouse model	SS-31 restores myocardial morphology and function, suppresses inflammation and oxidative stress, maintains mitochondrial membrane potential, attenuates ROS, reduces apoptosis and fibrosis, and inhibits NF-κB, p38 MAPK, calpain, and proteasome activities.	(11, 88, 89)
	Polyphenolic antioxidants	Resveratrol	CLP sepsis mouse/rat model	Resveratrol improves cardiac function, attenuates mitochondrial damage, upregulates PGC-1α, and inhibits ferroptosis via Sirt1/Nrf2 pathway activation.	(90, 91)
		Curcumin	CLP sepsis rat model; LPS-induced sepsis mouse model	Curcumin improves cardiac function and survival, promotes cardiomyocyte survival and mitochondrial biogenesis via SIRT1 activation, inhibits Drp1-mediated fission, downregulates TLR1/NF-κB signaling, and alleviates SIC.	(92, 93)
	Nrf2 activators	Sulforaphane	CLP sepsis mouse model	Sulforaphane reduced cardiac injury markers, decreased TLR4/NF-κB signaling, and oxidative stress, increased Bcl-2 and IL-10, and attenuated cardiac tissue injury via MD-2 competitive binding.	(94)
		Mangiferin	LPS-induced sepsis mouse model	Mangiferin enhanced survival and cardiac function via Nrf2 activation, protected mitochondrial integrity, prevented mtDNA release, and inhibited the cGAS-STING pathway.	(95)
	Melatonin		LPS-induced sepsis mouse model	Melatonin inhibited Ripk3, improved mitochondrial function, reduced ROS and ER stress, protected the cytoskeleton, and activated cardioprotective pathways; Ripk3 overexpression blocked these effects.	(43)
Mitochondrial dynamics regulators	DRP1 inhibitor	Mdivi-1	CLP sepsis mouse model; LPS-induced sepsis mouse model	Mdivi-1 inhibits Drp1, prevents mitochondrial fission and MOMP, preserves ATP, reduces ROS and Cyt C release, improves cardiac function, and suppresses NLRP3 inflammasome activation.	(96–99)
	P110 peptide		LPS-induced sepsis mouse model	P110 selectively blocks the Drp1/Fis1 interaction, reduces mitochondrial fragmentation, improves complex I/III activities, prevents mPTP opening, and attenuates myocardial injury with minimal off-target effects.	(34)
Mitochondrial Biogenesis Activators	PGC-1α activators	AICAR	CLP sepsis mouse/rat model	AICAR, an AMPK agonist, indirectly activates PGC-1α to enhance mitochondrial biogenesis, increase mtDNA copy number, upregulate TFAM and NRF1/2, improve cardiac ATP content, and promote autophagic flux for damaged mitochondria clearance.	(100–102)
	Resveratrol		Doxorubicin-treated mice	Resveratrol activates SIRT1-PGC-1α signaling to increase mitochondrial mass, upregulate OXPHOS complex subunits, and enhance fatty acid oxidation in septic cardiomyocytes.	(103)
Targeted interventions based on mitophagy inhibition	Caspase-1 inhibitor	VX765	ApoE^-/-^ and Ldlr^-/-^ mice with an atherosclerosis model	VX765 inhibits the caspase-1/NLRP3 inflammasome, promotes Parkin-mediated mitophagy and M2 polarization, mitigates mitochondrial damage, and alleviates inflammation in a Parkin-dependent manner.	(40)
	Tat-Beclin-1 peptide		LPS-induced sepsis mouse model	Beclin-1 promotes autophagy and PINK1-Parkin-mediated mitophagy, suppresses mTOR signaling, improves cardiac function, and rescues deficiency phenotypes.	(41)
	Traditional Chinese medicine	Po-Ge-Jiu-Xin Decoction	CLP sepsis rat model	Po-Ge-Jiu-Xin Decoction activates PINK1/Parkin-mediated mitophagy to reduce mortality, ameliorate myocardial injury, and alleviate mitochondrial dysfunction; these effects are partially reversed by Parkin siRNA.	(42)
Inflammation-mitochondria axis modulators	MCC950		CLP sepsis rat model	MCC950 inhibits NLRP3 inflammasome activation, reduces IL-1β/IL-18, and myocardial inflammation, prevents pyroptosis, and preserves mitochondrial function; hepatotoxicity limits clinical development.	(104)
	Cyclosporine A		CLP sepsis rat model;	CsA inhibits mPTP opening via cyclophilin D binding, preserves mitochondrial integrity, prevents DAMPs release, and improves cardiac function; the narrow therapeutic window and immunosuppressive effects limit its use, with NIM811 investigated as a safer alternative.	(105, 106)
	H-151		CLP sepsis mouse model	H-151 inhibits the cGAS-STING pathway, prevents mtDNA-driven type I interferon responses, and reduces cardiac inflammation in preclinical sepsis models.	(107)

AICAR, 5-Aminoimidazole-4-carboxamide ribonucleotide; AMPK, AMP-activated protein kinase; ApoE^−/−^, Apolipoprotein E knockout; ATP, Adenosine triphosphate; Bcl-2, B-cell lymphoma 2; cGAS-STING, Cyclic GMP-AMP synthase-stimulator of interferon genes; CLP, Cecal ligation and puncture; CsA, Cyclosporine A; Cyt C, Cytochrome c; DAMPs, Damage-associated molecular patterns; Drp1, Dynamin-related protein 1; ER, Endoplasmic reticulum; EUK-8, Eukaryotic cell-derived superoxide dismutase/catalase mimetic 8; Fis1, Fission 1; IL-1β, Interleukin-1 beta; IL-6, Interleukin-6; IL-10, Interleukin-10; IL-18, Interleukin-18; Ldlr^−/−^, Low-density lipoprotein receptor knockout; LPS, Lipopolysaccharide; MAPK, Mitogen-activated protein kinase; MD-2, Myeloid differentiation factor 2; Mdivi-1, Mitochondrial division inhibitor 1; mPTP, Mitochondrial permeability transition pore; MOMP, Mitochondrial outer membrane permeabilization; mtDNA, Mitochondrial DNA; mtROS, Mitochondrial reactive oxygen species; NF-*κ*B, Nuclear factor kappa-B; NLRP3, NOD-like receptor family pyrin domain containing 3; Nrf2, Nuclear factor erythroid 2-related factor 2; Nrf1/2, Nuclear respiratory factor 1/2; OXPHOS, Oxidative phosphorylation; P110, Drp1/Fis1 interaction blocking peptide; PGC-1α, Peroxisome proliferator-activated receptor gamma coactivator 1-alpha; PINK1, PTEN-induced putative kinase 1; Ripk3, Receptor-interacting serine/threonine-protein kinase 3; ROS, Reactive oxygen species; SIC, Sepsis-induced cardiomyopathy; SIRT1, Sirtuin 1; SOD, Superoxide dismutase; SS-31, Elamipretide; STING, Stimulator of interferon genes; TNF-α, Tumor necrosis factor alpha; TLR4, Toll-like receptor 4; TLR9, Toll-like receptor 9; TFAM, Mitochondrial transcription factor A; TPP⁺, Triphenylphosphonium cation; Vit-E, Vitamin E.

### 4.1 Mitochondria-Targeted antioxidants in the treatment of SIC

#### 4.1.1 Lipophilic cation-conjugated antioxidants

##### 4.1.1.1 Mitochondria-targeted coenzyme Q10 (MitoQ)

MitoQ, formed by covalently linking CoQ10 with the triphenylphosphonium cation (TPP⁺), represents a mitochondria-targeted antioxidant that selectively accumulates in the mitochondrial inner membrane. Lowes et al. (10) demonstrated in a LPS-peptidoglycan rat model of sepsis that MitoQ treatment significantly reduced biochemical markers of acute hepatic and renal dysfunction, maintained mitochondrial membrane potential across multiple organs, and suppressed proinflammatory cytokine release while promoting the production of the anti-inflammatory factor IL-10. Supinski et al. (37) further revealed that MitoQ prevented endotoxin-induced mitochondrial dysfunction in the myocardium. Through Langendorff perfusion techniques and polarographic measurements, this study demonstrated that MitoQ reversed endotoxin-induced reductions in mitochondrial state 3 respiration rates, respiratory control ratio, and ATP generation, while simultaneously improving cardiac pressure-generating capacity. The underlying mechanism involved the inhibition of caspase-9 and caspase-3 activation, thereby reducing cardiomyocyte apoptosis. These findings indicate that MitoQ protects mitochondrial structure and function, effectively ameliorating sepsis-induced myocardial contractile dysfunction.

##### 4.1.1.2 Mitochondria-targeted vitamin E (Mito-Vit-E)

Yao et al. (81) employed Mito-Vit-E in a pneumonia-related sepsis model to elucidate the critical role of the mtROS-mtDNA damage-inflammatory signaling pathway in SIC. The study found that Mito-Vit-E significantly protected mtDNA integrity, reduced mtDNA damage, and maintained expression levels of mtDNA repair enzymes, including DNA polymerase *γ* and AP endonuclease. More importantly, Mito-Vit-E inhibited mtDNA leakage into the cytoplasm, thereby blocking the upregulation of TLR9 pathway key factors MYD88 and receptor for advanced glycation end-products, subsequently suppressing NF-*κ*B and caspase-1 activation, reducing inflammasome component ASC expression, and decreasing IL-1β production. Ultimately, these effects led to reduced circulating cardiac troponin I levels, ameliorated cardiomyocyte apoptosis, and improved cardiac morphology. Zang et al. (82) further confirmed through echocardiographic analysis that Mito-Vit-E significantly improved cardiac contractile function in septic animals, increasing left ventricular fractional shortening and ejection fraction, with superior efficacy compared to non-targeted vitamin E, fully demonstrating the advantages of the mitochondria-targeted strategy.

SOD mimetics represent another important class of mitochondria-targeted antioxidant strategies, demonstrating unique value in the prevention and treatment of SIC. Tempol, a piperidine nitroxide compound, is a membrane-permeable SOD mimetic that efficiently scavenges superoxide anions. Al-Kadi et al. (83) discovered in a cecal ligation and puncture (CLP) sepsis model that Tempol treatment increased rat survival from 12.5% to 75%, significantly improving cardiac and renal function. The mechanism involved upregulating Klotho protein expression, inhibiting the p38-MAPK pathway, reducing TNF-α and caspase-3 expression, while restoring SOD activity and glutathione levels. Notably, this study first revealed the novel mechanism by which Tempol exerted cardiac protection through regulating Klotho, an anti-aging protein. Additionally, the salen-manganese complex EUK-8 possesses dual SOD and CAT activities. It was confirmed that EUK-8 dose-dependently attenuates cardiac and renal injury and circulatory failure in endotoxic shock (84).

##### 4.1.1.3 Vitamin C

Although vitamin C itself is not a mitochondria-targeted molecule, it holds important significance in SIC treatment as a water-soluble antioxidant. Shati et al. (85) found that vitamin C pretreatment significantly reduced myocardial malondialdehyde (MDA) levels, restored SOD activity, and decreased inflammatory markers, including TNF-α and IL-6, in septic rats. Light and electron microscopy observations confirmed its structural protective effects on cardiomyocytes, intercalated disks, mitochondria, and endothelial cells. Notably, combined application of vitamin C and vitamin E produces synergistic effects. Rinne et al. (86) observed in isolated cardiomyocyte experiments that simultaneous application of both vitamins demonstrated marked overadditive protective effects against singlet oxygen-induced oxidative damage compared to single agent application, suggesting that combined antioxidant strategies may be more effective.

##### 4.1.1.4 Combination therapeutic strategies

Based on the complementary mechanisms of different antioxidants, researchers have proposed multi-target combination therapeutic strategies. Marik et al. (87) pioneered the combination of intravenous vitamin C, hydrocortisone, and thiamine in patients with severe sepsis and septic shock. Results demonstrated that this regimen significantly reduced hospital mortality, shortened vasopressor usage duration, and prevented progressive organ dysfunction. This “metabolic resuscitation” strategy simultaneously intervened in multiple pathways, including oxidative stress, corticosteroid insufficiency, and mitochondrial metabolic disorders, providing novel insights for comprehensive treatment of SIC.

In conclusion, mitochondria-targeted antioxidants demonstrate promising application prospects in experimental research and clinical practice for SIC through multiple mechanisms, including the precise elimination of mtROS, protection of mtDNA and membrane structural integrity, and inhibition of inflammatory signaling pathways. Future research should further optimize timing, dosage, and targeting strategies and conduct large-scale randomized controlled trials to validate clinical efficacy.

#### 4.1.2 Peptide-based mitochondrial antioxidants

Peptide-based mitochondria-targeted antioxidants represent a novel class of therapeutic agents that have demonstrated promising cardioprotective effects in SIC. Unlike lipophilic cation-conjugated antioxidants that accumulate in mitochondria driven by membrane potential, these peptide compounds utilize a distinct targeting mechanism based on their cationic charge and selective affinity for the mitochondrial inner membrane, enabling them to exert protective effects independently of mitochondrial membrane potential. This characteristic may confer particular advantages in sepsis, where mitochondrial membrane potential is frequently compromised.

##### 4.1.2.1 Szeto-schiller 31 (SS-31)

SS-31, also known as elamipretide or Bendavia, is the most extensively studied peptide-based mitochondria-targeted antioxidant. This small tetrapeptide contains alternating aromatic and basic amino acids that facilitate its selective accumulation within the mitochondrial inner membrane. SS-31 exerts its protective effects primarily through scavenging mtROS, stabilizing cardiolipin, and maintaining mitochondrial membrane potential.

Liu et al. (11) provided the first direct evidence that SS-31 ameliorates sepsis-induced myocardial dysfunction in a LPS-induced murine model. In this study, C57BL/6 mice received intraperitoneal LPS administration to induce systemic inflammation and SIC. The investigators demonstrated that SS-31 treatment significantly restored myocardial morphological architecture and suppressed inflammatory responses, as evidenced by marked reductions in *IL-6*, *IL-1*β, and *TNF-*α mRNA levels in both *in vitro* and *in vivo* settings. Furthermore, SS-31 effectively ameliorated myocardial energy deficiency secondary to sepsis, normalized the activities of key antioxidant enzymes, including MDA, glutathione peroxidase, and SOD, and maintained mitochondrial membrane potential. Mechanistically, western blot analysis revealed that the cardioprotective effects of SS-31 were partially mediated through modulation of the NF-*κ*B signaling pathway, with concomitant inhibition of p38 MAPK, JNK1/2, and ERK phosphorylation. These findings established that SS-31 therapy effectively protected the heart against LPS-induced cardiac damage through dual mechanisms of oxidative stress attenuation and inflammatory response suppression.

The p38 MAPK signaling pathway appears to play a central role in SS-31-mediated cardioprotection. Another study investigated SS-31 effects in doxorubicin-induced cardiotoxicity and demonstrated that the peptide exerted cardioprotective effects by inhibiting p38 MAPK activation, attenuating ROS levels, stabilizing mitochondrial membrane potential, and ameliorating myocardial apoptosis and fibrosis (88). The specificity of this mechanism was confirmed using P79350, a selective p38 MAPK agonist, which reversed the protective effects of SS-31. Given the shared pathophysiological features between doxorubicin cardiotoxicity and SIC, including mitochondrial oxidative stress and p38 MAPK activation, these findings supported the applicability of SS-31 in sepsis-induced cardiac dysfunction.

While direct studies of SS-31 in SIC remain limited, investigations in related sepsis-induced muscle dysfunction provide additional supportive evidence. Supinski et al. (89) examined SS-31 effects in a CLP model of sepsis-induced diaphragm dysfunction. This study demonstrated that SS-31 completely prevented sepsis-induced reductions in diaphragm force generation. SS-31 also preserved diaphragm endurance, inhibited calpain and 20S proteasomal activities, maintained myosin heavy chain levels, and restored mitochondrial function as evidenced by improved state 3 respiration rates and ATP generation. Notably, SS-31 increased mitochondrial aconitase activity, indicating reduced mitochondrial superoxide generation. These findings in the diaphragm muscle, which shared physiological and pathological characteristics with cardiac muscle in sepsis, suggested that SS-31 may exert similar protective effects in SIC through preservation of mitochondrial function and prevention of proteolytic degradation.

#### 4.1.3 Other types of mitochondria-targeted antioxidants

##### 4.1.3.1 Polyphenolic antioxidants

Polyphenolic antioxidants, particularly resveratrol and curcumin, demonstrate significant cardioprotective effects in SIC through diverse molecular mechanisms targeting oxidative stress, inflammation, and mitochondrial dysfunction. Smeding et al. (90) demonstrated that resveratrol significantly improved myocardial contractile function and attenuated mitochondrial ultrastructural damage in a CLP murine model, with upregulation of PGC-1*α* modulating bioenergy metabolism and oxidative stress pathways. Zeng et al. (91) further revealed that resveratrol attenuates SIC through inhibition of ferroptosis via the Sirt1/Nrf2 pathway, with effects comparable to the specific ferroptosis inhibitor Ferrostatin-1. Chen et al. (92) identified TLR1 as a key molecular target for curcumin, which bound stably to TLR1 through hydrogen bonding, downregulating TLR1 expression and inhibiting NF-*κ*B phosphorylation to increase cardiomyocyte survival. Another research demonstrated that curcumin activates SIRT1, increasing mitochondrial biogenesis-related genes while reducing DRP1 translocation to inhibit mitochondrial fission, with SIRT1 knockdown reversing these protective effects. Both polyphenols activate SIRT1/PGC-1*α* signaling to promote mitochondrial biogenesis and inhibit NF-*κ*B-mediated inflammation, though bioavailability limitations and the discrepancy between functional improvement and survival outcomes necessitate further investigation for clinical translation (93).

##### 4.1.3.2 Nrf2 activators

Sulforaphane, a natural isothiocyanate derived from cruciferous vegetables, has demonstrated direct cardioprotective effects in experimental sepsis models. Hadi et al. (94) showed in a CLP murine model that sulforaphane significantly attenuated cardiac injury by reducing TLR-4, IL-6, TNF-α, F2-isoprostane, caspase-3, cardiac troponin I, and creatine kinase-MB levels while upregulating the anti-apoptotic protein Bcl-2, with its protective mechanism attributed to suppression of TLR-4/NF-*κ*B downstream signal transduction pathways and competitive binding to myeloid differentiation factor-2 (MD-2) that inhibits LPS interaction with the TLR4/MD-2 complex. Other natural Nrf2 activators have shown similar promise: mangiferin protects mitochondrial membrane integrity and suppresses cGAS-STING pathway-related inflammation (95), astilbin activated the NRF2/HO-1 pathway while inhibiting TLR4/NF-*κ*B signaling to reduce ventricular fibrillation susceptibility, and crocetin improved LPS-induced abnormalities in mitochondrial respiration and fatty acid *β*-oxidation through the p65/Keap1 and Nrf2/HO-1/NQO1 pathways (108). These studies suggest that different Nrf2 activation strategies may exert cardioprotection through distinct mechanisms ranging from direct mitochondrial and anti-inflammatory effects to systemic hemodynamic improvements.

##### 4.1.3.3 Melatonin

Melatonin has emerged as a promising therapeutic agent for SIC through multifaceted protective effects targeting mitochondrial function, ER homeostasis, and programmed cell death pathways. In a LPS-induced murine model, melatonin significantly attenuated septic myocardial injury by inhibiting Ripk3 expression, with protective effects comparable to genetic Ripk3 depletion (43). Mechanistically, melatonin improved mitochondrial bioenergetics by restoring ATP content, oxygen consumption rate, and mitochondrial membrane potential while reducing ROS production and enhancing antioxidant enzyme activities; additionally, it sustained mitochondrial dynamics, ameliorated ER stress, normalized calcium recycling, protected contractile cytoskeleton integrity, and activated cardioprotective signaling pathways while suppressing injury signals. The essential role of Ripk3 was confirmed by overexpression experiments, which rendered cardiomyocytes resistant to melatonin therapy. Furthermore, PIK3CG has been identified as an additional critical target, with molecular docking demonstrating that melatonin forms stable interactions with the PIK3CG protein to modulate myocardial inflammation, oxidative stress, and apoptosis. Collectively, these findings establish melatonin as a potent therapeutic candidate for SIC through Ripk3-dependent and PIK3CG-mediated mechanisms that restore the physiological functions of mitochondria, the ER, and the contractile apparatus.

### 4.2 Mitochondrial dynamics regulators

Excessive mitochondrial fission and suppressed fusion represent a central pathogenic mechanism in SIC, driving mitochondrial network fragmentation, bioenergetic collapse, and cardiomyocyte death (34, 35) Targeted modulation of mitochondrial dynamics offers a promising therapeutic strategy to restore morphological and functional integrity.

#### 4.2.1 Mdivi-1 (DRP1 inhibitor)

Mdivi-1 is a selective small-molecule inhibitor of Drp1 GTPase activity that blocks Drp1 translocation to the OMM and prevents oligomerization, thereby inhibiting pathological mitochondrial fission. In LPS-induced SIC models, Mdivi-1 pretreatment significantly attenuated mitochondrial fragmentation, preserved ATP synthesis capacity, reduced ROS generation, and improved cardiac contractile function (96–98). Mechanistically, Mdivi-1 prevented Drp1/Fis1 interaction and subsequent MOMP, thereby reducing Cyt C release and caspase-3 activation. Additionally, Mdivi-1 preserved Mfn1/2 and OPA1 expression levels, indirectly promoting fusion capacity. Notably, Mdivi-1 also exhibited anti-inflammatory effects by suppressing NLRP3 inflammasome activation and IL-1β maturation, suggesting dual protective mechanisms targeting both mitochondrial dynamics and inflammation (99).

#### 4.2.2 P110 peptide

P110 is a selective peptide inhibitor of the Drp1/Fis1 interaction that specifically blocks the binding interface between Drp1 and its mitochondrial receptor Fis1 without affecting Drp1 basal activity. Unlike Mdivi-1, which broadly inhibits Drp1 GTPase activity, P110 offers more targeted intervention by selectively disrupting the pathological fission complex without interfering with physiological mitochondrial division. In preclinical sepsis models, P110 administration reduced mitochondrial fragmentation, improved complex I and III activities, and attenuated myocardial injury markers (34). The peptide also preserved mitochondrial calcium handling capacity and prevented mPTP opening, thereby reducing necrotic and apoptotic cell death. Importantly, P110 demonstrated favorable safety profiles with minimal off-target effects on other dynamin family GTPases.

Collectively, mitochondrial dynamics regulators represent a rapidly evolving therapeutic class for SIC. By restoring the fission-fusion balance, these agents not only improve mitochondrial morphology but also enhance OXPHOS efficiency, reduce ROS production, and prevent inflammatory amplification. Future development should focus on improving pharmacokinetic properties and validating optimal timing of administration relative to disease onset.

### 4.3 Mitochondrial biogenesis activators

Mitochondrial biogenesis, the process of generating new mitochondria through coordinated nuclear and mitochondrial gene expression, offers a complementary strategy to antioxidant and dynamics-based approaches. In SIC, where mitochondrial content and function are severely compromised, promoting biogenesis may restore cellular bioenergetic capacity and support long-term metabolic recovery.

#### 4.3.1 PGC-1*α* activators

PGC-1*α* serves as the master regulator of mitochondrial biogenesis, coordinating the expression of genes involved in OXPHOS, fatty acid oxidation, and antioxidant defense. 5-Aminoimidazole-4-carboxamide ribonucleotide (AICAR), an AMPK agonist, indirectly activates PGC-1*α* through AMPK-mediated phosphorylation. In LPS-induced sepsis models, AICAR treatment enhanced mitochondrial biogenesis, increased mtDNA copy number, and improved cardiac ATP content (100–102). The mechanism involved AMPK-dependent PGC-1*α* activation and subsequent upregulation of mitochondrial transcription factor A (TFAM) and nuclear respiratory factors (NRF1/2). Additionally, AICAR promoted autophagic flux, facilitating the clearance of damaged mitochondria alongside the generation of new organelles. However, AICAR's poor oral bioavailability and potential to induce lactic acidosis limit its clinical applicability, necessitating the development of more selective AMPK activators or direct PGC-1*α* modulators.

#### 4.3.2 Resveratrol

Resveratrol, a natural polyphenol, activates SIRT1-mediated deacetylation of PGC-1*α*, thereby promoting mitochondrial biogenesis. As discussed in Section 5.1.3, resveratrol has demonstrated cardioprotective effects in SIC through multiple mechanisms (90, 91). Specifically regarding biogenesis, resveratrol increased mitochondrial mass, upregulated OXPHOS complex subunits, and enhanced fatty acid oxidation capacity in septic cardiomyocytes (103). These effects were mediated through SIRT1/PGC-1*α*/TFAM signaling cascade and were associated with improved cardiac output and survival in CLP models. The dual capacity of resveratrol to simultaneously activate biogenesis and inhibit excessive fission makes it a particularly attractive multi-target candidate for SIC therapy.

Mitochondrial biogenesis activators may prove especially valuable in the recovery phase of SIC, when restoration of mitochondrial content is essential for long-term cardiac function. However, their efficacy depends critically on the presence of functional mtDNA templates and intact transcriptional machinery, which may be compromised in severe or prolonged sepsis. Therefore, biogenesis-based strategies may be most effective when combined with agents that protect existing mitochondrial components, such as antioxidants or dynamics regulators.

### 4.4 Targeted interventions based on mitophagy modulation

As detailed in Section 3.6.2, targeted interventions based on mitophagy modulation delay SIC progression by regulating autophagy-inflammasome interactions. The therapeutic candidates including VX765, Tat-Beclin-1 peptide, PHB1 modulators, and Po-Ge-Jiu-Xin Decoction have demonstrated cardioprotective efficacy through distinct molecular mechanisms. Future development should focus on optimizing delivery systems and validating stage-specific administration protocols to maximize therapeutic benefit.

### 4.5 Inflammation-Mitochondria axis modulators

The bidirectional crosstalk between mitochondrial dysfunction and inflammatory activation constitutes a self-amplifying vicious cycle that perpetuates cardiac injury in SIC. Interrupting this axis at critical nodes offers a strategic approach to break the cycle and enable mitochondrial recovery.

#### 4.5.1 MCC950

MCC950 is a potent and selective small-molecule inhibitor of the NLRP3 inflammasome that blocks both canonical and non-canonical inflammasome activation. In experimental sepsis models, MCC950 administration significantly reduced IL-6 and IL-8 maturation, attenuated myocardial inflammatory infiltration, and improved survival rates (104). The cardioprotective mechanism involves direct inhibition of NLRP3 oligomerization and ASC speck formation, thereby preventing caspase-1 activation and subsequent pyroptosis. Importantly, MCC950 also indirectly preserves mitochondrial function by preventing mtDNA and cardiolipin-driven inflammasome amplification, reducing the inflammatory burden on damaged organelles. Unlike broad-spectrum anti-inflammatory agents, MCC950 specifically targets the NLRP3 pathway without affecting other innate immune responses, potentially offering a more favorable safety profile. However, clinical development of MCC950 has been complicated by hepatotoxicity concerns, prompting investigation of structurally related analogs with improved therapeutic indices.

#### 4.5.2 Cyclosporine A

Cyclosporine A (CsA) is a well-established immunosuppressant that also exhibits potent cardioprotective effects through inhibition of the mPTP. In SIC, CsA binds to cyclophilin D, a critical regulatory component of mPTP, preventing pore opening and subsequent mitochondrial swelling, membrane rupture, and DAMPs release (105, 106). By preserving mitochondrial membrane integrity, CsA prevents the release of mtDNA, cardiolipin, and Cyt C that drive inflammatory cascade activation. Preclinical studies demonstrated that CsA administered early in sepsis reduced cardiac troponin I levels, preserved left ventricular ejection fraction, and attenuated histological evidence of myocardial injury. The therapeutic window for CsA appears narrow, with efficacy declining when administration is delayed beyond 6 h post-injury, consistent with the temporal progression of irreversible mPTP opening. Concerns regarding immunosuppressive effects have limited enthusiasm for CsA in septic patients; however, non-immunosuppressive cyclophilin D inhibitors such as NIM811 are under investigation as potentially safer alternatives.

Additional inflammation-mitochondria axis modulators warrant consideration. The cGAS-STING pathway inhibitor H-151 prevents mtDNA-driven type I interferon responses and has shown promise in reducing cardiac inflammation in preclinical sepsis models (107). TNF-α inhibitors, though clinically disappointing in broad sepsis trials, may retain value in SIC subpopulations with documented elevated TNF-α levels. The key challenge for this therapeutic class lies in precise patient stratification: because inflammation-mitochondria crosstalk varies temporally and across individuals, biomarker-guided selection of candidates for axis modulation will be essential for successful translation.

## 5 Clinical translation challenges

Despite compelling efficacy demonstrated in animal models such as CLP and LPS-induced endotoxemia, mitochondria-targeted antioxidants and other promising sepsis therapies have consistently failed to translate into successful human clinical trials. This translational gap represents one of the most significant challenges in sepsis research and necessitates critical examination of the fundamental disconnect between preclinical models and human disease.

### 5.1 Biological complexity and timing of intervention

As delineated in Section 4.1, mitochondrial alterations follow a characteristic temporal sequence: early-phase metabolic reprogramming and modest ROS signaling may initially serve adaptive functions, whereas late-stage bioenergetic collapse, mitochondrial fragmentation, and cell death become irreversible (109). Early intervention risks disrupting beneficial stress responses, whereas delayed administration yields minimal efficacy (110).

The optimal timing for mitochondrial-targeted therapy remains poorly characterized in clinical practice. Preclinical studies typically administer agents prophylactically or within 1–2 h of insult, a scenario rarely achievable in human sepsis where diagnosis and treatment initiation frequently occur 6–12 h after organ dysfunction onset (110). Furthermore, the transition point from adaptive to pathological mitochondrial dysfunction varies substantially across individuals due to genetic background, comorbidity burden, and infection source heterogeneity. This variability precludes a universal treatment algorithm and necessitates dynamic biomarker-guided decision-making to identify the precise therapeutic window for each patient (111).

### 5.2 Lack of reliable biomarkers for patient stratification

Current clinical practice relies on indirect indicators such as lactate levels, the Sequential Organ Failure Assessment (SOFA) scores, and cardiac troponin I, none of which specifically reflect mitochondrial functional status (112). Serum mtDNA levels have emerged as a promising candidate, correlating with disease severity and NLRP3 inflammasome activation; however, standardized assays, reference ranges, and confounding factors remain unresolved (113).

Mitochondrial function assessment at the bedside remains technically challenging. *Ex vivo* measurement of mitochondrial respiration in peripheral blood mononuclear cells or platelets requires specialized equipment and expertise, limiting widespread applicability (114). Advanced imaging modalities such as magnetic resonance spectroscopy for cardiac ATP/phosphocreatine ratios show promise but are not yet integrated into routine critical care. Without reliable biomarkers to stratify patients by mitochondrial dysfunction phenotype and monitor treatment response, clinical trials cannot effectively enroll appropriate candidates or assess therapeutic efficacy, contributing substantially to translational failures (115).

### 5.3 Drug delivery and mitochondrial targeting efficiency

Even when optimal timing and patient selection are achieved, drug delivery and mitochondrial targeting efficiency remain formidable obstacles. Many mitochondria-targeted agents, including lipophilic cation-conjugated antioxidants and peptide-based compounds, exhibit poor tissue penetration due to high plasma protein binding, rapid renal clearance, and limited distribution into cardiac tissue (116). The TPP⁺ moiety that drives mitochondrial accumulation of MitoQ and MitoTempol also increases lipophilicity and biliary excretion, resulting in suboptimal bioavailability and requiring high dosing that may produce off-target effects (117).

Mitochondrial targeting specificity presents additional challenges. In sepsis, collapse of mitochondrial membrane potential, the very gradient that drives accumulation of lipophilic cations, impairs drug delivery to the most dysfunctional organelles where intervention is most needed (118). Peptide-based agents such as SS-31 offer advantages through membrane potential-independent targeting, but their susceptibility to proteolytic degradation and limited oral bioavailability necessitate parenteral administration, complicating clinical deployment (119). Nanoparticle-based delivery systems and mitochondrial-penetrating peptides are under active investigation to improve targeting specificity and stability, though these technologies remain largely preclinical (120).

### 5.4 Heterogeneity of SIC pathophysiology

The heterogeneity and complexity of human sepsis stand as the primary obstacle to successful translation. Marshall (12) emphasized that the systemic inflammatory response is biologically complex and redundant, activated by both infectious and noninfectious triggers, with more than 100 randomized clinical trials failing to demonstrate survival benefits from immunomodulatory therapies. Dyson et al. (44) systematically analyzed why novel therapies effective in animal models fail clinically, identifying that animal models remain too heterogeneous regarding type of insult, duration, and supportive therapy to be representative of human sepsis. Specifically, thousands of preclinical trials over 5 decades have yielded only a handful of drugs with demonstrated clinical efficacy, highlighting fundamental flaws in current research paradigms.

Mitochondrial dysfunction in SIC does not occur in isolation but interacts with multiple concurrent pathological processes. Inflammation-driven microcirculatory impairment limits oxygen delivery to cardiomyocytes, exacerbating mitochondrial hypoxia independent of ETC dysfunction (121). Metabolic dysregulation, including insulin resistance, hyperglycemia, and altered substrate availability, modifies mitochondrial fuel utilization and therapeutic response (122). Concurrent renal and hepatic dysfunction alters drug metabolism and clearance, further complicating dosing optimization. This multifactorial complexity means that mitochondria-targeted monotherapy is unlikely to achieve substantial clinical benefit; instead, combination strategies addressing inflammation, metabolism, and hemodynamics simultaneously may be required (114).

Disease characteristics and patient populations differ profoundly between animal models and clinical sepsis. Animal studies typically employ young, healthy, genetically homogeneous rodents without comorbidities, whereas human sepsis predominantly affects elderly patients with multiple preexisting conditions, including cancer, atherosclerosis, diabetes, and chronic kidney disease (CKD) (46, 123). This discrepancy is critical because comorbidities fundamentally alter host immune responses and pathophysiological mechanisms. For example, two-stage mouse models combining CKD with CLP-induced sepsis demonstrated distinct pathophysiological mechanisms compared to sepsis in healthy animals, with soluble fms-like tyrosine kinase-1 improving survival only in CKD-sepsis mice but not in healthy septic mice (46). Furthermore, immunological differences between species are substantial: laboratory mice are immunologically naive, while humans have been exposed to diverse antigenic environments from early life, and leukocyte subpopulation distributions, cytokine kinetics, and organ-specific responses differ significantly between murine and human systems (124, 125).

Intervention timing represents another critical factor contributing to translational failure. In most animal studies, drug administration is initiated before or within a few hours after septic insult, whereas septic patients are seldom treated within such an early therapeutic window (46). This temporal disconnect is particularly relevant for mitochondria-targeted antioxidants, where early intervention during the initial oxidative stress burst may be essential for efficacy. The CLP model itself presents additional limitations regarding clinical translation: humans rarely die from the primary infectious focus due to rapid response teams applying imaging, antibiotics, and source control, whereas most CLP mice die within 24 to 48 h from untreated peritonitis and bacteremia (126). Properly treated CLP mice with antibiotics, fluids, and surgical source control achieve 100% survival with no sequelae, further highlighting the artificial nature of untreated animal models (126).

The design and reporting of preclinical studies also contribute to translational failure. Inconsistent experimental designs, a lack of standardization, and inadequate reporting practices hinder reproducibility and comparability across studies (127). The Minimum Quality Threshold in Pre-Clinical Sepsis Studies guidelines were developed to address these issues by recommending standardized reporting of treatment timing, supportive care, randomization, blinding, and outcomes. Additionally, publication bias favoring positive results may create overly optimistic impressions of therapeutic efficacy that cannot be replicated in rigorous clinical trials.

The specific case of anti-TNF-α therapy illustrates these translational challenges: highly promising results in LPS-induced septic animals failed to improve survival in human clinical trials, leading many investigators to question the validity of LPS models as predictors of the human response (128). Similarly, more than 60 clinical trials testing novel sepsis treatments have failed to yield benefit for humans, with clinicians citing unconstructive animal tests as a primary reason for these failures (129).

Future directions to bridge this translation gap include developing animal models with preexisting comorbidities to better mimic human patient populations, employing larger animal models such as porcine systems that better simulate human cardiovascular and immune features, and integrating clinical data and biospecimens to strengthen connections between preclinical and clinical research. Additionally, stratification systems targeting treatment toward patients most likely to benefit based on clinical and biochemical phenotypes may improve trial success rates. Only through addressing these fundamental disconnects between animal models and human disease can the promising therapeutic potential of mitochondria-targeted antioxidants for SIC be successfully realized in clinical practice. The major barriers hindering clinical translation of mitochondria-targeted therapies are summarized in Table 3.

**Table 3:** Major barriers to clinical translation of sepsis therapies, with focus on mitochondria-targeted approaches.

Barrier category	Specific issue	Model/Population	Consequence for translation	References
Animal model vs. human differences	Young, healthy, genetically homogeneous rodents without comorbidities	CLP/LPS-induced sepsis mouse/rat models	Preclinical efficacy does not predict human response	(46, 123)
Absence of comorbidities	Human sepsis affects elderly patients with cancer, diabetes, and CKD	Animal models without comorbidities	Altered immune responses and pathophysiological mechanisms	(46)
Species immunological differences	Laboratory mice are immunologically naive; humans have diverse antigen exposure	Mice vs. humans	Different leukocyte distributions and cytokine kinetics	(124, 125)
Intervention timing mismatch	Drug administration initiated before or within hours after septic insult in animals	Animal models	Septic patients are seldom treated within such an early window	(46)
CLP model limitations	Untreated peritonitis in mice; humans receive rapid source control	CLP mouse model	Artificial disease course; proper treatment achieves 100% survival	(126)
Disease heterogeneity	Human sepsis has heterogeneity and complexity, courses, and severity	Heterogeneous patient populations	Single therapy cannot cover all subtypes	(12)
Clinical trial failure record	More than 100 randomized clinical trials and over 60 novel sepsis treatments failed to show benefit	Septic patients	Disconnect between animal models and clinical outcomes	(129)

CKD, Chronic kidney disease; CLP, Cecal ligation and puncture; LPS, Lipopolysaccharide.

## 6 Conclusion

The evidence synthesized in this review underscores that mitochondrial pathology extends far beyond mere energy failure, encompassing a complex spectrum of interconnected mechanisms that perpetuate cardiac injury. The intricate interplay between OXPHOS impairment, aberrant mitochondrial dynamics, defective mitophagy, and inflammation-driven damage creates a self-amplifying cycle that proves remarkably resistant to conventional therapeutic interventions. Therapeutic strategies targeting mitochondria have yielded encouraging results in experimental models. Mitochondria-targeted antioxidants, peptides such as SS-31, and modulators of mitochondrial quality control demonstrate substantial cardioprotective potential. Yet the persistent gap between preclinical efficacy and clinical success demands critical reflection. The homogeneous, well-controlled environment of animal studies bears little resemblance to the chaotic, multifactorial reality of human sepsis, where age, comorbidities, and genetic heterogeneity profoundly influence disease trajectory and therapeutic response.

Future research must prioritize the development of more sophisticated animal models that faithfully replicate human disease complexity, incorporating preexisting conditions and advanced supportive care protocols. Stratification of patients based on specific mitochondrial dysfunction phenotypes may prove essential for identifying those most likely to benefit from targeted interventions. Integration of multi-omics approaches, real-time mitochondrial function monitoring, and dynamic assessment of metabolic status could transform our capacity to deliver precision medicine at the bedside.

Ultimately, successful therapeutic translation will require abandoning the reductionist pursuit of single targets in favor of comprehensive strategies that address the multifaceted nature of mitochondrial pathology. By embracing the complexity of SIC rather than circumventing it, we may finally bridge the divide between promising laboratory discoveries and meaningful improvements in patient survival.
